# Fractures in pituitary adenoma patients from the Dutch National Registry of Growth Hormone Treatment in Adults

**DOI:** 10.1007/s11102-016-0716-3

**Published:** 2016-04-05

**Authors:** N. C. van Varsseveld, C. C. van Bunderen, A. A. M. Franken, H. P. F. Koppeschaar, A. J. van der Lely, M. L. Drent

**Affiliations:** Department of Internal Medicine, Endocrine section, Neuroscience Campus Amsterdam, VU University Medical Center, P.O. Box 7057, 1007 MB Amsterdam, The Netherlands; Department of Internal Medicine, Isala Clinics, Zwolle, The Netherlands; Emotional Brain and Alan Turing Institute for Multidisciplinary Health Research, Almere, The Netherlands; Division of Endocrinology and Metabolism, Department of Internal Medicine, Erasmus Medical Center, Rotterdam, The Netherlands

**Keywords:** Growth hormone, Fractures, Growth hormone deficiency, Cushing’s disease, Acromegaly, Nonfunctioning pituitary adenoma, Bone

## Abstract

**Purpose:**

The effects of growth hormone (GH) replacement therapy on fracture risk in adult GH deficient (GHD) patients with different etiologies of pituitary GHD are not well known, due to limited data. The aim of this study was to investigate characteristics and fracture occurrence at start of (baseline) and during long-term GH replacement therapy in GHD adults previously treated for Cushing’s disease (CD) or acromegaly, compared to patients with previous nonfunctioning pituitary adenoma (NFPA).

**Methods:**

From the Dutch National Registry of Growth Hormone Treatment in Adults, a nationwide surveillance study in severe GHD adults, all patients using ≥30 days of GH replacement therapy with previous NFPA (n = 783), CD (n = 180) and acromegaly (n = 65) were selected. Patient characteristics, fractures and potential influencing factors were investigated.

**Results:**

At baseline, patients with previous CD were younger, more often female and had more often a history of osteopenia or osteoporosis, whereas patients with previous acromegaly had more often received cranial radiotherapy and a longer duration between treatment of their pituitary tumor and start of adult GH replacement therapy. During follow-up, a fracture occurred in 3.8 % (n = 39) of all patients. Compared to patients with previous NFPA, only patients with previous acromegaly had an increased fracture risk after 6 years of GH replacement therapy.

**Conclusions:**

During GH replacement therapy, an increased fracture risk was observed in severe GHD adult patients previously treated for acromegaly, but not in those previously treated for CD, compared to severe GHD adult patients using GH replacement therapy because of previous NFPA. Further studies are needed to confirm these findings and to elucidate potential underlying mechanisms.

## Introduction

Severe growth hormone deficiency (GHD) in adults is an increasingly recognized clinical entity, characterized by unfavorable alterations in body composition, cardiovascular risk factors and quality of life [1–3]. Moreover, adult GHD is associated with decreased bone turnover and bone mineral density (BMD) [4–7]. In addition, several studies have demonstrated a three- to fivefold increased fracture risk in GHD patients compared with healthy controls [6, 8–10]. Long-term growth hormone (GH) replacement therapy in these patients has shown a biphasic effect on bone turnover and BMD. After an initial increase in bone resorption, which may result in unchanged or decreased BMD, an increase in BMD is seen, which is sustained up to 15 years [4–6, 11–13]. Nevertheless, available literature regarding fractures, a clinically relevant endpoint, is limited [5, 6, 8–11, 13–17]. Compared with healthy controls, Holmer et al. [17] found an increased fracture risk in women with childhood onset GHD (CO-GHD) using GH replacement therapy, but a decreased fracture risk in men with adult onset GHD (AO-GHD) using GH replacement therapy. In another report, the prevalence of radiological vertebral fractures was higher in untreated GHD patients than in those treated with GH replacement therapy (78.6 vs. 53.8 %) [16]. In a recent large observational study, the annual clinical fracture incidence was significantly lower in adult GHD patients with GH replacement therapy than in those without GH replacement therapy (1.19 vs. 1.91 %) [14]. Moreover, in a prospective study including GHD adult patients, 30 % of the patients developed radiological vertebral fractures during 6 years of follow-up. Vertebral fracture incidence was related to pre-existing vertebral fractures at study entry and untreated GHD. GH replacement therapy led to a significant decrease in vertebral fracture risk [18]. Although these studies may point toward a beneficial effect of GH replacement therapy on fractures in GHD patients, they show a wide variation in study design, population, follow-up duration and endpoints. Moreover, the effect of GH replacement therapy on fractures in patients with specific causes of GHD has not been thoroughly investigated.

Various underlying disorders may lead to GHD, of which pituitary adenomas (PAs) and their treatments are the most common in adults [19]. These underlying disorders may influence the clinical presentation of GHD and possibly the response to GH replacement therapy [20]. For instance, Cushing’s disease (CD), a severe endocrine disorder characterized by hypercortisolism, is associated with, amongst others, osteoporosis and increased fracture risk [21–23]. Acromegaly, due to a GH-secreting PA, has also been related with abnormalities of the skeletal system [4–6, 24–27]. In a recent meta-analysis, skeletal fragility was found to be an emerging complication of acromegaly [25]. An increased risk of (vertebral) fractures has been described in some, but not all, studies, even after long-term control of acromegaly [5, 26, 28].

Few studies have compared bone related parameters between GHD patients previously treated for CD, acromegaly and other underlying causes of GHD [10, 20, 29–32]. In one study, bone markers and BMD were similar in patients with previous acromegaly and nonfunctioning pituitary disease during 2 years of GH replacement therapy [32]. In another study comparing GHD patients treated for CD and acromegaly, respectively, with those treated for other causes of GHD, a lower BMD and higher fracture prevalence were found in CD patients at baseline, but not in acromegaly patients [20]. However, the occurrence of fractures during GH replacement therapy was not specifically investigated.

Therefore, the aim of the present study was to investigate patient characteristics as well as occurrence of fractures and potential influencing factors in a large cohort of adult GHD patients with previous CD, acromegaly and nonfunctioning pituitary adenoma (NFPA), respectively, at the start of and during long-term GH replacement therapy. Using data from Dutch National Registry of Growth Hormone Treatment in Adults, a nationwide long-term surveillance study in severe GHD adult patients, GHD patients with previous CD and acromegaly were compared to those with previous NFPA.

## Materials and methods

### Study population

The Dutch National Registry of Growth Hormone Treatment in Adults was initiated by the Dutch Ministry of Health in 1998 to gain more insight into the long-term efficacy, safety and costs of GH replacement therapy in GHD adults. From that time on, reimbursement of GH replacement therapy costs was linked to approval of the indication, severe GHD, by an independent board of endocrinologists as well as entry of anonymous patient data into the registry. All patients were informed by their attending physician. Severe GHD was diagnosed according to the Growth Hormone Research Society consensus guidelines [33]. Until data closure in 2009, 2891 severe GHD adults were registered. Data collection, characteristics and test procedures of these patients have previously been described in more detail [34].

For this study, all patients with previous NFPA (n = 893), acromegaly (n = 72) and CD (n = 198) were selected. Patients who had received <30 days of GH replacement therapy were excluded (n = 110, n = 18 and n = 7 patients with previous NFPA, CD and acromegaly, respectively). Excluded patients (n = 135) had a similar age at entry into the registry (*p* = 0.17), gender (*p* = 0.99) and onset of GHD (*p* = 0.39), compared to patients included in the study (n = 1028). Patients who were lost to follow-up, stopped GH replacement therapy or died, were censored in the analyses.

### Measurements

Data of all registered patients were collected (bi-) annually from medical records by specially trained monitors from the start of enrollment in the registry. When GH replacement therapy had already been started before the first monitor visit, data were retrospectively retrieved. As an internal quality control, the data of 10 % of the patients were collected twice by different monitors. Anonymized collected data were checked for accuracy both before and after entry into the database.

Persons-years of treatment with GH replacement therapy were calculated from the date of commencement with GH replacement therapy in adulthood (baseline) until the date of last follow-up, discontinuation of GH replacement therapy or death. The GH dose was titrated on an individual basis by the attending physician with the purpose of achieving and maintaining age- and gender-specific normalized insulin-like growth factor 1 (IGF-1) SD scores. Changes in GH dosage were recorded and the mean dose per patient was calculated as the cumulative dosage divided by the sum of GH replacement therapy days.

The diagnosis of the pituitary tumor, i.e. NFPA, CD or acromegaly, was made at the discretion of the attending physician and verified at entry into the database according to the collected data. Data regarding the treatment of the tumor, such as surgical procedures, medication and radiotherapy, were collected. Other pituitary hormone deficiencies in addition to GHD were identified through recorded deficiencies and hormonal replacement therapies, based on diagnostic tests performed by the attending physicians, at the start of registration and during follow-up. These deficiencies were adequately substituted when appropriate. Calcium, bisphosphonate, vitamin D and other osteoporosis medication use were recorded at baseline and during follow-up.

Smoking status, alcohol use, height and weight, used to calculate body mass index (BMI, kg/m^2^), were collected from the start of entry into the registry.

Relevant medical history was searched thoroughly and coded for osteopenia/osteoporosis, diabetes and fractures. In a minority of patients, baseline dual X-ray absorptiometry (DXA) data were available. Several types of DXA scanners were used. Measured skeletal sites included the lumbar spine, femoral neck, trochanter, total hip and/or total body. According to widely used criteria, osteopenia was defined as a T-score between −1 and −2.5 and osteoporosis as a T-score equal to or below −2.5 at one of the measured sites [35]. In patients with available DXA data, these data were used to complement and verify reported osteopenia/osteoporosis.

Adverse events were recorded thoroughly and coded for fractures. All types of fractures were included. If >1 fracture occurred at the same time, the largest bone that was fractured was counted as the fractured bone. If >1 fracture occurred over time in the same patient, only the first fracture was included in the analysis. The date of fracture was that of the first recorded event.

### Statistical analysis

Continuous variables were expressed as either mean (SD) or median (range), while categorical variables were expressed as number and/or percentage. Parametric or nonparametric tests were used when appropriate.

Kaplan–Meier survival curves were used to describe the event-free survival. Cox proportional hazard analysis was used to investigate fracture risk. Time was measured from start of GH replacement therapy in adulthood until the date of first fracture, last follow-up or death, whichever occurred first. Assumptions of proportional hazards were tested by log-minus-log plots and interaction terms. A significant interaction with time was observed, resulting in different hazard ratio’s (HRs) before and after 6 years of follow-up.

To examine relevant confounding, potentially confounding variables (gender, age, history of fractures, history of osteopenia/osteoporosis, osteoporosis medication use, extent of pituitary insufficiency [isolated GHD or multiple pituitary hormone deficiencies], adrenocorticotropic hormone [ACTH] insufficiency, luteinizing hormone [LH]/follicle-stimulating hormone [FSH] insufficiency, pituitary surgery, radiotherapy and time between tumor treatment and start of GH replacement therapy) were added separately to the unadjusted model. Age and time between tumor treatment and start of GH replacement therapy, both continuous variables, were checked for linearity with the outcome variable and because of nonlinearity divided into categories. To examine potential effect modification by gender, an interaction term was added to the gender-adjusted model. In case of a *p* value <0.10 analyses were stratified for gender. All statistical analyses were performed using the statistical software package IBM SPSS Statistics version 20. Two-sided *p* values of ≤0.05 were considered significant.

## Results

### Baseline characteristics

Of the 1028 severe GHD adult patients included in the present study, 783 (76.2 %) had previous NFPA, 180 (17.5 %) previous CD and 65 (6.3 %) previous acromegaly. In 924 (89.9 %) patients, severe GHD was diagnosed with a GH stimulation test (53.2 % insulin tolerance test, 22.6 % Growth Hormone-Releasing Hormone [GHRH]/arginine test, 15.0 % arginine test, 8.8 % GHRH test, 0.3 % other). In 97 (9.4 %) patients, the diagnosis was based on serum IGF-1 concentrations ≤2 SD in combination with two or more additional pituitary hormone deficiencies, whereas in 7 (0.7 %) patients either retesting was considered unnecessary because of CO-GHD due to evident hypothalamic pituitary disease (n = 2 NFPAs), or the diagnostic procedure was unknown (n = 4 NFPAs and n = 1 CD).

Baseline characteristics of all patients are presented in Table 1. In 414 (40.3 %) patients, baseline DXA data were available. Of these patients, 71 (17.1 %) had osteoporosis at one or more of the measured sites (15.6, 26.4 and 11.8 % of patients with previous NFPA, CD and acromegaly, respectively, *p* = 0.06), whereas 147 (35.5 %) had osteopenia (34.4, 43.1 and 29.4 % of patients with previous NFPA, CD and acromegaly, respectively, *p* = 0.29). Combining these two categories, 218 (52.7 %) patients had a T-score below -1 (50.0, 69.4 and 41.2 % of patients with previous NFPA, CD and acromegaly, respectively, *p* = 0.004).

**Table 1:** Baseline characteristics of adult GHD patients with previous nonfunctioning pituitary adenoma, Cushing’s disease and acromegaly, respectively

	NFPA	CD	Acromegaly	*p* value^a^
No. of patients	783	180	65	
Age at baseline, years (mean, SD)	54.8 (11.6)	47.4 (12.7)	53.0 (11.7)	<0.001
Age at pituitary tumor diagnosis, years (mean, SD)	48.0 (13.2)	35.6 (12.9)	36.9 (11.9)	<0.001
Gender, female	305 (39.0)	122 (67.8)	38 (58.5)	<0.001
Pituitary surgery^b^	726 (92.8)	153 (85.0)	59 (90.8)	0.003
Cranial radiotherapy^c^	390 (50.2)	111 (61.7)	51 (78.5)	<0.001
Adrenalectomy^d^		65 (36.7)		
Extent of pituitary insufficiency				
IGHD	40 (5.1)	19 (10.6)	10 (15.4)	<0.001
ACTH insufficiency	601 (76.8)	137 (76.1)	44 (67.7)	0.26
TSH insufficiency	627 (80.1)	127 (70.6)	47 (72.3)	0.01
LH/FSH insufficiency	636 (81.2)	112 (62.2)	44 (67.7)	<0.001
ADH insufficiency	105 (13.4)	38 (21.1)	4 (6.2)	0.004
PRL insufficiency	5 (0.6)	3 (1.7)	1 (1.5)	0.34
≥3 other pituitary hormone deficits	487 (62.2)	90 (50.0)	33 (50.8)	0.004
Onset of GHD, childhood onset	5 (0.6)	4 (2.2)	0 (0.0)	0.09
Time between tumor treatment and start of GH replacement therapy, years (median, range)^e^	3.6 (−1.6 to 46.0)	8.7 (0.27–48.6)	14.8 (1.2–47.8)	<0.001
Medical history^f^				
Osteopenia or osteoporosis (T-score < −1)^g^	199 (25.9)	80 (45.2)	20 (30.8)	<0.001
Fractures	109 (14.2)	28 (15.8)	8 (12.3)	0.76
Diabetes	66 (8.6)	19 (10.7)	1 (1.5)	0.08
Smoking^h^				0.02
Yes	185 (28.0)	39 (26.9)	14 (48.3)	
Former	168 (25.6)	25 (18.3)	5 (17.2)	
No	304 (46.3)	81 (55.9)	10 (34.5)	
Alcohol use^i^	246 (46.5)	44 (36.4)	9 (36.0)	0.09
BMI, kg/m^2^ (mean, SD)^j^	28.6 (4.8)	28.2 (5.4)	29.6 (5.7)	0.25
Osteoporosis medication^k^				
Calcium	64 (8.2)	28 (15.6)	8 (12.5)	0.008
Bisphosphonates	30 (3.8)	18 (10.1)	8 (12.5)	<0.001
Vitamin D	37 (4.7)	14 (7.8)	5 (7.8)	0.18
Other	0 (0.0)	3 (1.7)	2 (3.1)	<0.001
Dopamine agonists	26 (3.3)	2 (1.1)	4 (6.2)	0.11
Ketoconazole	0 (0.0)	3 (1.7)	0 (0.0)	<0.001
Gonadal replacement therapy				
Males	239 (50.0)	20 (34.5)	13 (48.1)	0.08
Females	102 (33.4)	34 (27.9)	6 (15.8)	0.06

*GHD* growth hormone deficiency, *NFPA* nonfunctioning pituitary adenoma, *CD* Cushing’s disease, *IGHD* isolated growth hormone deficiency, *ACTH* adrenocorticotropic hormone, *TSH* thyrotrophin, *LH* luteinizing hormone, *FSH* follicle-stimulating hormone, *ADH* antidiuretic hormone, *PRL* prolactin, *GH* growth hormone, *BMI* body mass index

^a^Continuous variables were tested with one-way ANOVA or Kruskal–Wallis test. Categorical variables were examined with the Chi square test

^b–d^Missing subjects: ^b^ n = 1; ^c^ n = 6; ^d^ n = 3

^e^Time from surgery or primary radiotherapy for the pituitary adenoma or first confirming MRI when no surgery or radiotherapy was initiated until start of GH replacement therapy

^f^Missing subjects: n = 17

^g^Based on available DXA data and other data from medical records

^h–k^Missing subjects: ^h^ n = 198; ^i^ n = 353; ^j^ n = 283; ^k^ n = 5

### Follow-up characteristics

Median follow-up time was 5.2 (0.1–20.2) years, representing 4065 person-years of GH replacement therapy, for patients with previous NFPA, 6.1 (0.2–15.8) years, corresponding with 1104 treatment-years, for patients with previous CD and 3.1 (0.2–13.7) years, being 280 treatment-years, for patients with previous acromegaly (*p* < 0.001). Daily median GH dose differed between the three groups (0.27 [0.04–1.26] vs. 0.31 [0.10–1.45] vs. 0.28 [0.10–0.83] mg in the NFPA, CD and acromegaly group, respectively, *p* < 0.001).

During follow-up, osteoporosis medication was used by 166 (21.3 %) of the patients with previous NFPA, 69 (38.5 %) of the patients with previous CD and 22 (33.4 %) of the patients with previous acromegaly (*p* < 0.001). This included use of calcium (17.3 vs. 31.8 vs. 21.9 % of patients with previous NFPA, CD and acromegaly, respectively, *p* < 0.001), bisphosphonates (11.5 vs. 25.1 vs. 20.3 % of patients with previous NFPA, CD and acromegaly, respectively, *p* < 0.001), vitamin D (12.3 vs. 21.8 vs. 14.1 % of patients with previous NFPA, CD and acromegaly, respectively, *p* < 0.001) and other osteoporosis medication (0.6 vs. 4.5 vs. 4.7 % of patients with previous NFPA, CD and acromegaly, respectively, *p* < 0.001).

### Fractures

During follow-up, 39 (3.8 %) patients (fracture data not available in one patient) had a fracture, including 26 (3.3 %) patients in the NFPA group, 8 (4.4 %) patients in the CD group and 5 (7.8 %) patients in the acromegaly group. The majority of the patients (n = 32 [82.1 %]) had one fracture. Median time between baseline and first fracture during follow-up was 2.4 (0.04–10.0) years, being 1.9 (0.04–9.6), 3.0 (0.9–8.7) and 8.3 (0.5–10.0) years for patients with previous NFPA, CD and acromegaly, respectively. Mean age at time of first fracture during follow-up was 58.7 (11.1) years. Most first fractures (n = 15 [38.5 %]) were located in the lower or upper arm (also including wrist and clavicle), followed by the hip (n = 5 [12.8 %]), foot (n = 5 [12.8 %]) and tibia/fibula (n = 4 [10.3 %]). Vertebral fractures were reported in 2 (5.1 %) cases (both in the NFPA group). Characteristics of patients with and without a fracture during follow-up are shown in Table 2.

**Table 2:** Characteristics of patients with and without a fracture during follow-up

	No fracture during follow-up	Fracture during follow-up	*p* value^a^
No. of patients	988	39	
Age at baseline (mean, SD)	53.3 (12.1)	54.9 (11.7)	0.42
Age at pituitary tumor diagnosis, years (mean, SD)	45.2 (14.0)	43.9 (14.2)	0.55
Gender, female	441 (44.6)	24 (61.5)	0.04
Pituitary surgery^b^	903 (91.5)	34 (87.2)	0.38
Cranial radiotherapy^c^	525 (53.4)	26 (68.4)	0.07
Adrenalectomy^d^	60 (35.5)	5 (62.5)	0.15
Extent of pituitary insufficiency			
IGHD	65 (6.6)	4 (10.3)	0.33
ACTH insufficiency	750 (75.9)	31 (79.5)	0.61
TSH insufficiency	772 (78.1)	29 (74.4)	0.58
LH/FSH insufficiency	766 (77.5)	25 (64.1)	0.05
ADH insufficiency	139 (14.1)	8 (20.5)	0.26
PRL insufficiency	9 (0.9)	0 (0.0)	1.00
≥3 other pituitary hormone deficits	587 (59.4)	23 (59.0)	0.96
Onset of GHD, childhood onset	9 (0.9)	0 (0.0)	1.00
Time between tumor treatment and start of GH replacement therapy, years (median, range)^e^	4.4 (−1.6 to 48.6)	7.0 (0.7 to 38.4)	0.03
Medical history^f^			
Osteopenia or osteoporosis (T < −1)^g^	276 (28.4)	22 (56.4)	<0.001
Fractures	135 (13.9)	10 (25.6)	0.04
Diabetes	82 (8.4)	4 (10.3)	0.57
BMI, kg/m^2^ (mean, SD)^h^	28.6 (5.0)	28.7 (4.8)	0.93
Osteoporosis medication during follow-up^i^			
Calcium	191 (19.4)	14 (35.9)	0.01
Bisphosphonates	137 (13.9)	10 (25.6)	0.04
Vitamin D	138 (14.0)	6 (15.4)	0.81
Other	14 (1.4)	2 (5.1)	0.12
Gonadal replacement therapy during follow-up			
Males	186 (42.2)	8 (33.3)	0.39
Females	382 (69.8)	11 (73.3)	1.00

*IGHD* isolated growth hormone deficiency, *ACTH* adrenocorticotropic hormone, *TSH* thyrotrophin, *LH* luteinizing hormone, *FSH* follicle-stimulating hormone, *ADH* antidiuretic hormone, *PRL* prolactin, *GHD* growth hormone deficiency, *GH* growth hormone, *BMI* body mass index

^a^Continuous variables were tested with one-way ANOVA or Kruskal–Wallis test. Categorical variables were examined with the Chi square test

^b–d^Missing subjects: ^b^ n = 1; ^c^ n = 6; ^d^ n = 3

^e^Time from surgery or primary radiotherapy for the pituitary adenoma or first confirming MRI when no surgery or radiotherapy was initiated until start of GH replacement therapy

^f^Missing subjects: n = 17

^g^Based on available DXA data and other data from medical records

^h–i^Missing subjects: ^h^ n = 283; ^i^ n = 5

**Fig. 1:**
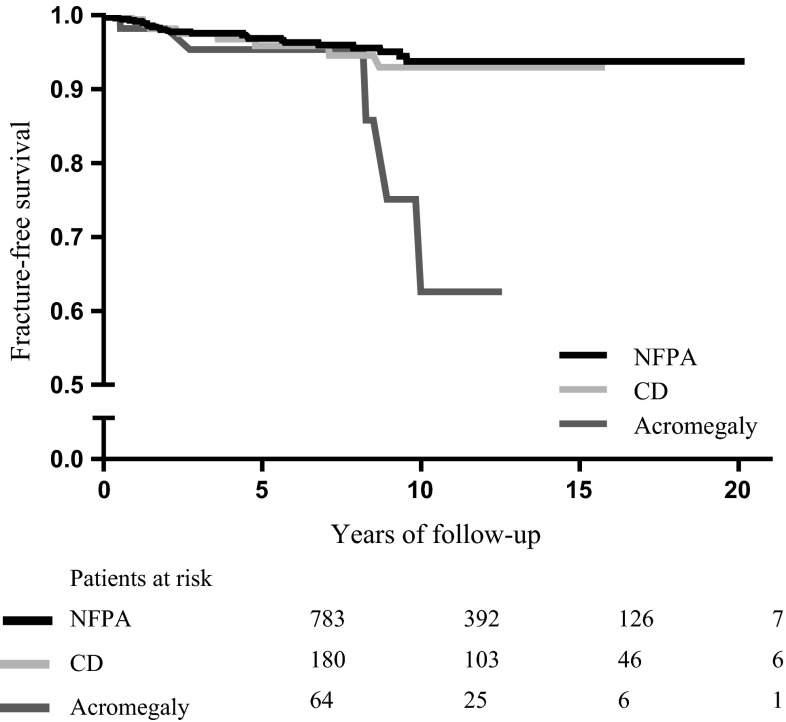
Kaplan–Meier curve showing the fracture-free survival of adult GHD patients with previous nonfunctioning pituitary adenoma, Cushing’s disease and acromegaly, respectively, using GH replacement therapy. *Legend* *GHD* growth hormone deficiency, *GH* growth hormone, *NFPA* nonfunctioning pituitary adenoma, *CD* Cushing’s disease

The fracture-free survival of patients with previous NFPA, CD and acromegaly is shown in Fig. 1. In Cox proportional hazard analysis a significant interaction with time was observed (*p* = 0.05), resulting in different HRs before and after 6 years of follow-up (Table 3). Before 6 years of follow-up, fracture risk did not differ between patients with previous NFPA, CD and acromegaly, whereas after 6 years, fracture risk was increased in patients with acromegaly, but not in those with CD, compared to patients with NFPA. Adjustment for potential confounders, including gender, age, history of fractures, history of osteopenia/osteoporosis, osteoporosis medication use, extent of pituitary insufficiency, ACTH insufficiency, LH/FSH insufficiency, pituitary surgery, radiotherapy and time between tumor treatment and start of GH replacement therapy did, not substantially influence the results (data not shown). Until 6 years of follow-up, 29 of 579 (5.0 %) patients had a fracture, including 21 (4.7 %) patients in the NFPA group, 6 (6.7 %) in the CD group and 2 (4.7 %) in the acromegaly group. After 6 years of follow-up, 10 of 448 (2.2 %) patients had a fracture, including 5 (1.5 %), 2 (2.2 %) and 3 (14.3 %) patients in the NFPA, CD and acromegaly groups, respectively.

**Table 3:** Results of Cox proportional hazard analysis for fracture risk in adult GHD patients with previous nonfunctioning pituitary adenoma, Cushing’s disease and acromegaly, respectively, using GH replacement therapy

		Fractures			
	NFPA	CD		Acromegaly	
		HR (95 % CI)	*P* value	HR (95 % CI)	*p* value
Unadjusted model					
<6 years follow-up	Reference	1.28 (0.51–3.16)	0.61	1.60 (0.37–6.89)	0.53
≥6 years follow-up	Reference	1.31 (0.25–6.76)	0.75	12.06 (2.88–50.61)	<0.001

*GHD* growth hormone deficiency, *GH* growth hormone, *NFPA* nonfunctioning pituitary adenoma, *CD* Cushing’s disease, *HR* hazard ratio

Analyses were stratified for follow-up time (lowest *P* value interaction terms for time 0.05)

## Discussion

The present observational study of severe GHD adult patients using GH replacement therapy, included a large cohort of patients with previous NFPA, CD and acromegaly, respectively. Characteristics of these patients at initiation of and during adult GH replacement therapy were investigated with the focus on fractures and possible contributing factors. Compared to patients with previous NFPA, patients with previous acromegaly, but not those with previous CD, appeared to have an increased fracture risk after 6 years of adult GH replacement therapy. Before 6 years of follow-up, however, no increased fracture risk was observed.

At baseline, patients with previous CD were, amongst others, younger, more often female and had more often a history of osteopenia or osteoporosis, whereas patients with previous acromegaly had a longer duration between tumor treatment and start of adult GH replacement therapy and were more often treated with radiotherapy. Similar differences have also been observed in other studies [20, 30, 31].

The higher occurrence of osteopenia or osteoporosis in the medical history of previous CD patients is in accordance with other reports of lower BMD in patients with (treated) CD [20, 23, 29, 30]. This is probably due to the well-recognized long-term effects of hypercortisolism on bone [20, 22, 29, 31, 36, 37]. It is assumed that glucocorticoids mainly affect bone metabolism directly by decreasing osteoblast number and functioning, resulting in suppression of bone formation [6, 22, 37, 38]. Although strong improvements in BMD and bone architecture have been reported after successful treatment of hypercortisolism, recovery may be incomplete or may take years [22, 36, 37]. Moreover, in treated CD patients with concomitant GHD, there may be a combined detrimental effect of previous longstanding hypercortisolism and GHD on bone [20, 29].

In patients with active CD, the estimated fracture rate ranges between 15 and 50 % [22, 37]. In two reports from the KIMS database, the prevalence of fractures prior to start of GH replacement therapy was higher in GHD patients treated for CD than in patients with other etiologies of GHD [20, 30]. In contrast, in our database fractures were not observed more frequently in patients with previous CD. In another study, comparing patients with Cushing’s syndrome with healthy controls, fracture risk was only increased in the last 2 years prior to diagnosis of Cushing’s syndrome and reduced to normal after diagnosis and treatment [23]. This is in line with our results, as most of our CD patients had been diagnosed and treated for CD years before start of adult GH replacement therapy. Also, it has been postulated that GHD patients with the lowest baseline BMD may have the greatest response to GH replacement therapy [11–13, 29]. As osteopenia or osteoporosis were most frequently recorded in the history of our CD patients, it may be that these patients had the greatest benefit of GH replacement therapy with regard to bone. Possibly, GH replacement therapy ameliorates the damaging effects of glucocorticoid excess on the skeleton [39]. In addition, the use of osteoporosis medication at baseline and during follow-up was higher in the CD group, which may also have decreased fracture risk.

Fractures occurred in 3.8 % of the patients during follow-up. Although adult GHD is associated with decreased bone turnover and lower BMD, data on fracture risk are scarce in literature, as most studies have used BMD, but not fractures, as the endpoint [4, 6, 7, 12]. In adult GHD patients without GH replacement therapy, the prevalence of fractures appears to be increased compared to non-GHD controls [8–10]. GH replacement therapy induces an initial increase in bone resorption, followed by a sustained increase in BMD after at least 1 year of GH replacement therapy [5–7, 11–13]. A decreased fracture risk in a subgroup of men with AO-GHD using GH replacement therapy compared to population controls was reported in one study [17]. Another study demonstrated a significantly higher prevalence of radiological spinal deformities in untreated GHD patients compared to treated patients, suggesting a protective effect of GH replacement therapy [16]. Recently, the Hypopituitary Control and Complication Study (HypoCCS) reported a significantly lower annual fracture incidence in adult GHD patients with GH replacement therapy than in those without GH replacement therapy [14]. Of the patients with GH replacement therapy, 5.5 % had at least one fracture during follow-up, which is similar to the low frequency observed in our study. Likewise, in a Swedish cohort of GHD patients treated with GH replacement therapy up to 15 years, only two fractures were reported [11]. Although this might suggest an overall low fracture rate in adult GHD patients using GH replacement therapy, higher rates have also been described in other studies [8, 10, 16–18]. Comparison and interpretation of studies may be difficult due to differences in study design (mainly cross-sectional), follow-up duration, differences in causes of GHD, severity of GHD, age, other potential confounders and endpoints, e.g. (radiological) vertebral or nonvertebral fractures.

Evaluation of vertebral fractures may be difficult and prone to underestimation, especially in large observational studies such as the present study, which mainly depend on clinical assessment of vertebral fractures. It has been suggested that only one-quarter of radiologically identified vertebral fractures are clinically recognized by patients or physicians at the time of their occurrence [40, 41]. In a recent prospective study of community-dwelling older men, less than 15 % of incident radiographic vertebral fractures were also clinically diagnosed [42]. Therefore, identification of incident vertebral fractures on spinal radiographs, thereby using a standardized approach to assess changes in vertebral body shape and height, is considered a better method to evaluate vertebral fractures [5, 40]. In several reports by Mazziotti et al. [16, 18, 39, 43, 44] a quantitative morphometric approach was used to investigate radiological vertebral fractures in GHD adults. In a recent prospective study by Mazziotti et al., prevalent vertebral fractures were observed in 13 of 40 (32.5 %) adult GHD patients, while incident vertebral fractures occurred in 30 % of the patients. Additionally, GH replacement therapy had a beneficial effect on vertebral fracture risk [18]. Although a standardized imaging approach is considered the best way to assess vertebral fractures, this may unfortunately not be feasible in large observational studies based on daily clinical practice.

An interesting finding in the present study was the increased fracture risk after 6 years of follow-up in patients with previous acromegaly. As our study is one of the first to evaluate fracture occurrence in a clinical setting in adult patients with specific underlying etiologies of pituitary GHD, more research is awaited to further elucidate this finding. During active acromegaly, increased bone turnover has been observed, but reported effects on BMD are heterogeneous [4–6, 10, 24, 25]. It is postulated that cortical BMD increases, whereas trabecular BMD decreases or remains unaffected [4, 6, 24, 25]. In one study, a decreased fracture risk was observed in acromegalic patients [28]. However, a growing number of publications report an increased vertebral fracture risk [5, 25, 26]. This risk appears to be unrelated to BMD and may persist after long-term disease control, which is in accordance with findings in the present study [5, 25, 26].

Data on bone health, particularly fractures, in patients with GHD after treatment for acromegaly are limited [20, 32, 45]. In a report from the KIMS database, BMD and fracture prevalence did not differ between GHD patients with previous acromegaly and those with other etiologies of GHD [20]. The increased fracture risk in the present study may be a long-term effect of impaired skeletal health due to previous GH excess, even though this was not reflected by an increased occurrence of osteopenia or osteoporosis in the medical history. Indeed, the predictive value of BMD for fractures in acromegaly has been questioned [5, 6, 25, 26]. BMD may be overestimated by DXA due to frequently occurring degenerative skeletal changes in acromegaly [5, 25, 26]. Also, fracture risk may be more influenced by bone quality than bone quantity [5, 6, 26, 46]. Possibly, bone quality is irreversibly altered in patients with previous acromegaly [26, 47]. In addition, as a U-shaped curve has been suggested between fracture risk and GH concentration, patients who have experienced GH excess as well as GH deficiency may have a combined deleterious effect on their bone health [5]. Furthermore, other factors that have been associated with skeletal impairments and altered GH concentrations, such as gonadal status, other pituitary insufficiencies, gender, age, muscle strength and disease duration, probably also play a role [5, 6, 16, 17, 26]. Duration between pituitary tumor treatment and start of adult GH replacement therapy was significantly longer in patients with previous acromegaly than in those with previous NFPA and CD. However, additional adjustment for this factor did not materially change the results. Overall, although the number of events was low, our data may suggest that GH replacement therapy does not fully restore bone health in GHD patients with previous acromegaly. Potential underlying mechanisms seem to be complex and require further investigations [4–6, 25, 26, 45].

An important strength of the present study is the large population of severe GHD adult patients with specific underlying diagnoses, i.e. NFPA, CD and acromegaly, included. Other studies often included a mixture of patients with various causes of GHD, excluded patients with previous CD or acromegaly, or did not specifically evaluate skeletal health in these patients. In the present study, follow-up duration was considerate and several potentially confounding variables were evaluated. Also, thorough registration of events was ensured through (bi-) annual monitoring by specially trained nurses. Nevertheless, there are several limitations, inherent to the observational design of the study. Results could not be compared with a control group of untreated GHD patients. Ethical constraints, combined with the low incidence of GHD and fractures, impede the performance of such a randomized controlled trial. Also, the number of fractures was low, which could have influenced results. Furthermore, radiological assessment of fractures was not systematically performed. Therefore, the number of fractures, especially asymptomatic vertebral fractures, has probably been underestimated. In addition, DXA data were not available in all patients and different types of scanners were used. Nevertheless, our data represent daily clinical practice and as such provide valuable information.

In conclusion, the present study is one of few reports in literature evaluating fracture occurrence in severe GHD adult patients using GH replacement therapy with specific etiologies of GHD, i.e. NFPA, CD and acromegaly. An increased fracture risk was only observed in patients with previous acromegaly after 6 years of GH replacement therapy, but not in those with previous CD, compared to patients with previous NFPA. Although potential underlying mechanisms remain to be clarified, our data suggest that severe GHD patients previously treated for acromegaly may have an increased risk of fractures, possibly due to combined detrimental effects of previous GH excess as well as GH deficiency on bone. Further investigations are needed to confirm and elucidate these possible associations.
